# Does Analgesic Effect of Opium Hamper the Adverse Effects of Severe Coronary Artery Disease on Quality of Life in Addicted Patients?

**DOI:** 10.5812/aapm.5139

**Published:** 2012-07-10

**Authors:** Mahdi Najafi, Mehrdad Sheikhvatan

**Affiliations:** 1 Anesthesiology Department, Tehran Heart Center, Tehran University of Medical Sciences (TUMS), Tehran, Iran; 2 Research Department, Tehran Heart Center, Tehran University of Medical Sciences (TUMS), Tehran, Iran

**Keywords:** Coronary Artery Disease, Quality of Life, Opium, Analgesia

## Abstract

**Background:**

Opium is a unique substance, regarding its analgesic effects. This may change the deteriorating effects of coronary artery disease (CAD) on quality of life (QOL) in addicted patients.

**Objectives:**

We studied the QOL in opium-addicted and non-addicted CAD patients so as to determine the relationship between CAD risk factors and the subscales of their QOL.

**Patients and Methods:**

Demographic and laboratory data as well as coronary artery risk factors were obtained and SF-36 questionnaire was completed through interviews with 268 (38 opium-addicted and 230 non-addicted) patients with CAD who were candidates for isolated coronary artery bypass at Tehran Heart Center.

**Results:**

Mean Euro SCORE in addicted and non-addicted patients were 3.7 ± 7.6 and 2.4 ± 2.2 respectively (*P* = 0.036). In addicted group, higher preoperative HbA1c was associated with low physical function score (β = −0.395, *P* = 0.021). Low ejection fraction could negatively affect the general health (β = 0.394, *P* = 0.014) and mental health (β = 0.292, *P* = 0.015) subscales in the addicted group.

**Conclusions:**

Despite higher rate of morbidities in opium-addicted patients compared to non-addicted ones, subscales of QOL were similar between the two groups. High preoperative HbA1c and low ejection fraction appeared to be determinants of poor QOL in the opium-addicted patients.

## 1. Background

The problems associated with the use of purified agents such as opiates have attracted the attention of health and legal authorities as well as the general public (1). A significant amount of opium is still consumed in many regions of the world especially in Asia (2). It is estimated that the prevalence of opium addiction in Iran is 2–2.8% according to official statistics (3). In addition, the incidence of opium addiction is especially higher in the subgroups of some diseases, therefore the prevalence of substance dependence is between 9.9% and 19% in coronary artery disease (CAD) patients undergoing coronary revascularization (4, 5).

As uncontrolled pain may have deleterious physical and mental effects, it seems that controlling pain by opiates can affect the complications and quality of life (QOL) in patients who have severe untreatable diseases (6). Opioid therapy can relieve pain and improve mood and physical function in patients with chronic pain. This has led experts on pain to recommend not depriving such patients from opioids despite some reported complications (7, 8).

Based on known criteria of American psychiatric association, DSM IV-TR, addiction to opium dependence or addiction is a syndrome involving compulsive use of opium with tolerance and withdrawal. This is different from substance use which is defined as problematic use-not compulsive- without tolerance and withdrawal.

The impact of opium addiction on QOL status is questioned. Some trials of opium using have reported secondary outcome measures of QOL (9–11). Results from such clinical trials illustrated that the effects of opium addiction on QOL vary widely and QOL is found rather poor in these studies.

There is a paucity of information on the risks and benefits of opium use in perioperative period and any effect on QOL. As opium has especial analgesic effects in comparison with other substances, assessment of its impact on QOL in those undergoing major surgeries such as cardiac revascularization is necessary.

## 2. Objectives

The present study came to address the QOL status in opium addicted CAD patients. Besides, we wanted to know whether analgesic effects of opium improve the addicts’ QOL in the presence of cardiac risk factors or not.

## 3. Patients and Methods

In this study, 268 patients with the final diagnosis of CAD and candidates for isolated coronary artery bypass graft (CABG) in Tehran Heart Center were recruited in a period of 5 months. Any patient who had operations other than CABG, patients who were unable to complete psychological test and patients who were reluctant to perform an interview were excluded from the study. Among these, 38 patients were addicted to opium and 230 patients were not addicted. A data manager proposed the SF-36 questionnaire to the patients on admission to the surgical ward. The questionnaire was completed through an interview before the operation. The SF-36 is composed of eight subscales, namely physical functioning, role emotional, role physical, bodily pain, social functioning, mental health, vitality, and general health (12). The patients were also given self-administered questionnaires about their medical history and early complications after surgery.

The following data were included for analysis: the preoperative variables:

1. General characteristics: Age, gender, body mass index (BMI), and education level (primary education defined as primary school or less; secondary education characterized as secondary school level; and high education defined as university/college levels or equivalent) (13)
2. Preoperative risk factors: Current smoking history (patients regularly smoke a tobacco product/products one or more times per day or have smoked in the 30 days prior to admission) (14), alcohol abuse (repeated use despite recurrent adverse consequences) (15), opium dependence (according to the DSM IV-TR criteria for substance dependence in addition to having daily consumption) (16), hypercholesterolemia (total cholesterol ≥ 200 mg/dL, HDL-cholesterol ≤ 40 mg/dL in men, or ≤ 45 mg/dL in women, and triglycerides ≥ 180 mg/dL) (17), family history of CAD (first-degree relatives before the age of 55 in men and 65 years in women) (18), hypertension (systolic blood pressure ≥ 140 mmHg and/or diastolic ≥ 90 mmHg and/or on antihypertensive treatment) (19), diabetes mellitus (symptoms of diabetes plus at least one of the following: plasma glucose concentration ≥ 200 mg/dL, fasting plasma glucose ≥ 126 mg/dL, and 2-hpp ≥ 200 mg/dL) (20), cerebrovascular disease, and peripheral vascular disease
3. Preoperative cardiac status: Previous myocardial infarction (an acute event with abnormal creatine phosphokinase and troponin levels), Euro SCORE, and functional class
4. Preoperative homodynamic status: Number of coronary vessels involvements and left ventricular ejection fraction

Euro SCORE is a method of calculating predicted operative mortality for patients undergoing cardiac surgery. It stands for European system or cardiac operative risk evaluation. When patient risk factors are taken into consideration, operative mortality is a good measure to evaluate the quality of cardiac surgical care. If a risk factor is present in a patient, a weight or number is assigned. The weights are added to come up with an approximate predicted mortality. (For scoring details go to official site: http://euroscore.org/euroscore_scoring.htm).

We considered two criteria for a complicated postoperative short-term outcome:

1: In-hospital postoperative complications (existence of at least one of these complications: postoperative sustained arrhythmias, wound infection, and respiratory failure), and 2: In-hospital mortality rate (sometimes termed as operative mortality), defined as death in hospital after operation.

Having described the sample and its main characteristics, we explored the variations in QOL, subsequent to CABG and the predictors of these variations among addicted patients. Results were reported as mean ± standard deviation (SD) for the quantitative variables and categorical variables rate. The groups were compared using the student’s *t*-test for the continuous variables and the chi-square test (or Fisher’s exact test if required) or Mantel-Haenszel chi-square trend test for the categorical variables. The SF-36 scoring rules were applied to the questionnaire. The data analyzer was anonymous, and data collection and processing were approved by the institutional review board of our heart center. *P* values of 0.05 or less were considered statistically significant. All the statistical analyses were performed using SPSS version 13.0 (SPSS Inc., Chicago, IL, USA) and SAS version 9.1 for Windows (SAS Institute Inc., Cary, NC, USA).

## 4. Results

Demographic characteristics, preoperative clinical indices, and postoperative complications in the two groups are summarized in *Table 1*. Among general risk factors for CAD, cigarette smoking (*P* < 0.001) and recent myocardial infarction (*P* = 0.014) were significantly more prevalent in the addicted patients, whereas hyperlipidemia (*P* = 0.011) and diabetes mellitus (*P* = 0.033) were found more in the non-addicted ones. There were no significant differences in functional class and number of coronary vessels involvement between the two groups. The mean ejection fraction was lower (*P* = 0.002) and the mean Euro SCORE was higher (*P* = 0.036) among the addicted patients. Among common postoperative complications, arrhythmia (*P* = 0.007) and brain stroke (*P* = 0.002) were found more in the addicted patients, whereas respiratory failure, wound infection, and in-hospital mortality rates were similar.

**Table 1:** Comparison of Preoperative Characteristics and Postoperative Complications Between Opium-Addicted and Non-Addicted Patients

Characteristics	Opium-Addicted Patients, n = 38	Non-Addicted Patients, n = 230	*P* value
Male gender, No.	97.4	69.6	< 0.001
Mean age, y	56.0 ± 8.5	60.3 ± 8.9	0.006
BMI ^a^, kg/m^2^, mean ± SD	25.3 ± 3.7	27.5 ± 4.4	0.002
Education level, No.			0.033
Primary	36.8	54.3	
Secondary	34.2	32.1	
High	28.9	13.6	
Family history of CAD ^a^	55.3	44.3	0.211
Current cigarette smoking	84.2	30.0	< 0.001
Hyperlipidemia	50.0	70.9	0.011
Hypertension	36.8	52.2	0.080
Cerebrovascular disease	2.6	4.3	0.622
Diabetes mellitus	26.3	44.8	0.033
Peripheral vascular disease	15.8	21.7	0.403
Last creatinine, mmoL/L, mean ± SD	1.36 ± 0.23	1.29 ± 0.21	0.081
Previous myocardial infarction, No.	68.4	47.0	0.014
Ejection fraction, mean ± SD	44.6 ± 9.7	50.1 ± 9.6	0.002
Functional class, No.			0.758
I	36.8	33.0	
II	47.4	51.3	
III	15.8	15.7	
Euro score, mean ± SD	3.66 ± 7.64	2.36 ± 2.23	0.036
Number of defected vessels, No.			0.898
One	2.6	3.9	
Two	21.1	22.6	
Three	76.3	73.5	
Postoperative complications, No.			
Wound infection	2.6	0.8	0.314
Arrhythmias	55.3	32.8	0.007
Respiratory failure	21.1	14.3	0.287
Brain stroke	5.3	0.4	0.002

^a^Abbreviations: BMI, body mass index; CAD, coronary artery disease

The mean scores of SF-36 subscales in the addicted and non-addicted patients are depicted in *Table 2*. Among different subscales of QOL, only the mean of vitality was higher in opium-addicted versus non-addicted patients (*P* = 0.040).

**Table 2:** Comparison of SF-36 Subscales Between Opium-Addicted and Non-Addicted Patients

Characteristics	Opium-Addicted Patients, n = 38	Non-Addicted Patients, n = 230	*P* value
Physical functioning, mean ± SD	69.1 ± 24.1	64.8 ± 24.23	0.314
Role physical, mean ± SD	34.8 ± 37.9	35.5 ± 39.7	0.920
Bodily pain, mean ± SD	75.1 ± 27.8	71.5 ± 32.0	0.480
General health, mean ± SD	68.4 ± 17.2	69.8 ± 17.0	0.640
Vitality, mean ± SD	75.5 ± 18.9	68.3 ± 22.4	0.040
Social functioning, mean ± SD	75.8 ± 20.6	76.5 ± 25.8	0.849
Role emotional, mean ± SD	64.8 ± 43.1	61.6 ± 39.8	0.671
Mental health, mean ± SD	72.4 ± 19.6	66.6 ± 20.7	0.104

Among addicted patients, higher preoperative HbA1c was associated with low physical function score (β = −0.395, *P* = 0.021). Also, low ejection fraction could negatively affect the general health (β = 0.394, *P* = 0.014) and mental health (β = 0.292, *P* = 0.015) subscales in the addicted group.

## 5. Discussion

In the present study, we compared the patients’ demographic characteristics, history of CAD risk factors, and post-CABG complications between our opium-addicted and non-addicted patients before considering all the subscales of QOL in the two groups. We also assessed the relations between the patients’ characteristics and QOL subscales in the addicted group in order to determine the role that these characteristics play in QOL of opium-addicted CAD patients.

Considering general risk factors for CAD, addicted patients were typically men with lower age and higher rate of MI and cigarette smoking history, which are all regarded as determinants of poor outcome, supported by higher Euro SCORE mean in opium addicted patients (*Table 1*). Though higher age is usually considered as a risk factor for CAD, it may not be true in our patients who are young enough to be categorized as early CAD patients, known as true high risk population (21).

We found that among CAD risk factors, the history of hyperlipidemia and diabetes mellitus were more frequent and BMI was higher in non-addicted patients. It seems that the loss of weight is related to chronic suppression of appetite in opium-addicted patients, which may progress to an extreme degree, referred to as cachexia (22). So it’s not strange that patients with such low BMI have lower rate of diabetes type two and hyperlipidemia. However, poor dietary habits, predispose addicts to metabolic disturbances that threaten their health (23, 24).

With the focus on the cardiac problems, there was a higher cardiac arrhythmias rate and lower ejection fraction in addicted patients compared to non-addicts. It may be due to an increase in cardiac volumes and circulatory shortening rate of myocardial fibers, which suggest that the compensatory potentials of the myocardium are reduced in opium users (25).

In the present study, all the subscales of QOL were similar between the opium-addicted and non-addicted patients. To the best of our knowledge there are few, if any, studies on the relationship between opium addiction and QOL in CAD patients. The studies undergone among opiate and/or opioid abusers, are the closest to the current research. According to Bizzarri *et al.,* patients with opioid dependence showed significantly poorer QOL in the physical function, mental health, and social functions compared to healthy participants (9). Also in a study in Canada, opiate users mental and physical health were found worse than that of the general population (10). According to Smith *et al.*, physical function of adult substance abusers was similar to that of the other patients, but their mental health was much lower (11). However, it seems that memory impairment, mental slowing, and reduced motivation for purposeful activities other than those related to drug use are common symptoms in chronic heavy users.

The question is that why in our study, QOL in opium-addicted patients is not worse than that of their non-addict counterparts. Pain is the most annoying symptom in cardiac problems, and opioids are among the most important medications in angina pectoris treatment. So it’s possible that the analgesic effect of opium has decreased the adverse effects of chest pain with heart origin and resulted in reported higher QOL than expected in our addicted patients.

Ejection fraction was correlated with both mental and general health in the addicted patients. In Meyer study, improvement of ejection fraction due to walking indicated a significant inverse correlation with improvement in SF-36 mental subscale scores. Moreover, a significant correlation between improvement of peak power output after 12 weeks of rehabilitation and baseline physical sub-scale score was found (26). Previous studies have demonstrated that physical and mental subscales of QOL can be improved by exercise training and that rehabilitation program can result in an improvement of exercise capacity, ejection fraction, and dimensions of QOL (27).

HbA1c role in predicting outcome in CAD has been the focus of investigations in recent years (28). We found a relationship between HbA1c and physical function subscale of QOL. Though this is a novel finding, we need further study with larger sample size to judge the impact of HbA1c on QOL.

We also demonstrated that some general risk factors for CAD such as history of hyperlipidemia, myocardial infarction, and other characteristics related to the severity of CAD such as functional class, number of defected coronary arteries, and Euro SCORE did not influence any of eight QOL components in the opium-addicted patients. Be that as it may, an assessment of these factors and their relationships with QOL in a greater sample size is also needed.

In summary, although there were meaningful differences in preoperative characteristics and postoperative complications between the opium-addicted and non-addicted CAD patients, all the subscales of QOL were similar in the two groups. This may be explained by particular analgesic effect of opium which masks and/or attenuates the adverse effects of CAD on the patients. Furthermore, it looks like low ejection fraction and HbA1c are important predictors for QOL in the opium-addicted patients, which should be confirmed by further studies.
